# Local immune and microbiological responses to mucosal administration of a Liposome-TLR agonist immunotherapeutic in dogs

**DOI:** 10.1186/s12917-019-2073-8

**Published:** 2019-09-13

**Authors:** William Wheat, Lyndah Chow, Alana Kuzmik, Sirikul Soontararak, Jade Kurihara, Michael Lappin, Steven Dow

**Affiliations:** From the Center for Immune and Regenerative Medicine and the Center for Companion Animal Studies, Department of Clinical Sciences, College of Veterinary Medicine and Biomedical Sciences, Colorado State University, 80523, Ft. Collins, Colorado, CO USA

**Keywords:** Cytokine, T cells, Monocytes, Microbiome, Immunity, Immunotherapy, Infectious disease, Canine, Herpesvirus, Antimicrobial resistance, Liposomes, Toll-like receptor agonists

## Abstract

**Background:**

Non-specific immunotherapeutics have been evaluated previously in dogs, primarily for cancer treatment. However, there remains a need for a more broadly targeted, general purpose immunotherapeutic capable of activating innate immune defenses for non-specific protection or early treatment of viral and bacterial infections. To address need, our group has developed a liposomal immune stimulant (liposome-TLR complexes, LTC) containing TLR 3 and 9 agonists specifically designed to activate mucosal immune defenses in sites such as nasal cavity and oropharynx, following topical delivery. In this study, we evaluated the local immune stimulatory properties of LTC in vitro and in healthy purpose-bred dogs, including activation of cellular recruitment and cytokine production. The ability of LTC treatment to elicit effective antiviral immunity was assessed in dogs following a canine herpesvirus outbreak, and the impact of LTC treatment on the local microbiome of the oropharynx was also investigated.

**Results:**

These studies revealed that LTC potently activated innate immune responses in vitro and triggered significant recruitment of inflammatory monocytes and T cells into the nasal cavity and oropharynx of healthy dogs. Administration of LTC to dogs shortly after an outbreak of canine herpesvirus infection resulted in significant reduction in clinical signs of infection. Interestingly, administration of LTC to healthy dogs did not disrupt the microbiome in the oropharynx, suggesting resiliency of the microflora to transient immune activation.

**Conclusions:**

Taken together, these results indicate that LTC administration mucosally to dogs can trigger local innate immune activation and activation of antiviral immunity, without significantly disrupting the composition of the local microbiome. Thus, the LTC immune stimulant has potential for use as a non-specific immunotherapy for prevention or early treatment of viral and bacterial infections in dogs.

## Background

Dogs are susceptible to infection with a multitude of different viral and bacterial pathogens that cause respiratory tract infection and illness, including viral agents such as influenza, parainfluenza, and herpesviruses and bacterial pathogens such as *Bordetella* and mycoplasmas [1–3]. Though vaccines are available to prevent some of these infections, in cases where animals are crowded or stressed (e.g., boarding or day care facilities or airline flights) it may not be possible to vaccinate in time to prevent infection, or vaccine immunity may decline due to stress-induced immune suppression. With some pathogens, it is difficult to induce effective or durable immunity (e.g., *Bordetella canis*), and some pathogens may rapidly evolve to escape specific immunity (e.g., canine influenza [4]). For these reasons, there is currently a need for an immunotherapeutic capable of rapidly generating non-specific immune activation and protection from a diverse array of potential canine pathogens.

A number of immunotherapeutics have been developed and evaluated as potential cancer immunotherapeutics in dogs. For example, live *Mycobacterium bovis* and cell wall extracts from yeast and bacteria have all been evaluated for anti-tumor activity in dogs, typically following direct intra-tumor administration [5, 6]. Perhaps the best studied tumor immunotherapeutic has been the NOD like receptor agonist muramyl tripeptide (MTP), which has demonstrated impressive anti-tumor activity in multiple dog models [7–12]. Mechanistically, MTP immunotherapy was shown to activate macrophage activity and TNFα production in the lungs of treated animals [13–15]. Our laboratory has previously evaluated the use of liposome-TLR complexes (LTC) which potently activate type I innate immune responses, for immunological activity in dogs with several types of cancer, including metastatic osteosarcoma [16, 17]. Unlike the case with cancer immunotherapy, there are few non-specific immune stimulants with demonstrated activity against viral or bacterial pathogens in dogs.

We previously demonstrated in rodent infection models that cationic liposome-TLR complexes (LTC) containing non-coding plasmid DNA as a TLR9 agonist could potently activate innate immune responses and elicit highly effective protection against a variety of lethal viral and bacterial infections following mucosal administration of LTC via the intranasal route [18–23]. Moreover, we recently reported that LTC administered intranasally to cats could generate effective local immune activation and protection against FHV-1 [22, 24]. Therefore, we hypothesized that LTC could also generate effective prophylactic or early therapeutic immunity in dogs following mucosal administration. To address this question and evaluate feasibility of the new approach to infectious disease immunotherapy, we modified the original LTC to more specifically target mucosal immunity and to broaden the scope of innate immune activation, to include both TLR3 and TLR9 agonists.

In the present report, modified LTC [24] were evaluated for activation of innate immune responses in dogs, using both in vitro and in vivo assays. The studies focused on induction of local immune activation in the nasal cavity and oropharynx of dogs following intranasal administration of LTC to healthy Beagle dogs, and on whether such local immune activation could generate non-specific protection from viral infection. Finally, the impact of LTC administration on the microbiome of the oropharynx of dogs was investigated. Taken together, these studies provided convincing evidence that LTC potently activate local mucosal innate responses in the upper airways of dogs, accompanied by induction of non-specific anti-viral protective immunity.

## Results

### LTC administration triggers cellular activation of dog leukocytes in vitro

**Fig. 1:**
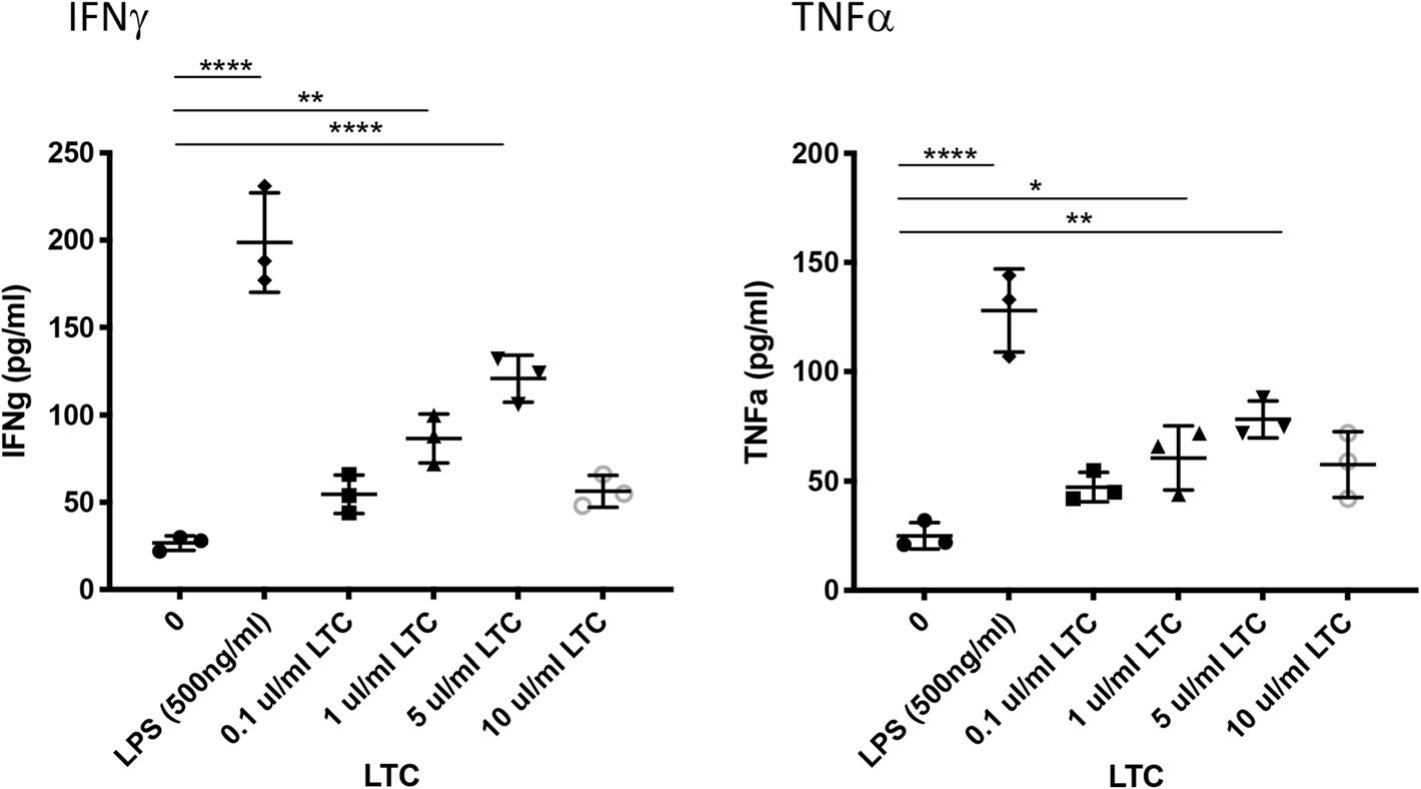
LTC treatment of dog leukocytes stimulates release of IFNγ and TNFα in a dose-dependent manner. Whole blood was obtained from healthy, purpose bred dogs (*n* = 3) and processed to generate peripheral blood mononuclear cells (PBMC) via density centrifugation through Ficoll. Cells were seeded in triplicate wells in complete tissue culture medium, as noted in Methods. The cells were then treated with the indicated amounts of LTC to activate cells and trigger cytokine production, which was monitored using specific canine ELISA assays for IFNγ and TNFα. Data were analyzed using one-way ANOVA with multiple comparisons. (*, *P* ≤ 0.05, **, *P* ≤ 0.01 ****, *P* ≤ 0.001). These results are representative of a total of 3 separate and independent experiments

To determine whether LTC treatment stimulated immune cell activation of canine leukocytes in vitro, PBMC were purified from whole blood and plated at 1 X 10^6^ cells/well and treated with several different concentrations of LTC to determine whether production of two key innate cytokines (IFNγ and TNFα) was upregulated. Supernatants were collected from PBMC cultures following 24 h stimulation with LTC, and IFNγ and TNFα concentrations were assessed by ELISA (Fig. 1). These studies revealed that LTC treatment stimulated significant dose-dependent, increased secretion of IFNγ and TNFα. It should also be noted that LTC doses ≥10 μl/ml decreased cytokine production, due to induction of cytotoxicity, which has been reported previously with liposome-TLR therapeutics [25].

### Macrophage treatment with LTC triggers TNFα production and upregulated expression of MHCII

**Fig. 2:**
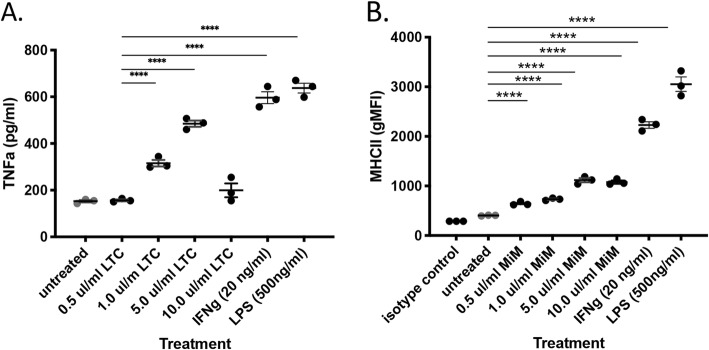
LTC treatment of dog macrophages stimulates TNFα production and upregulation of MHCII expression. Whole blood was obtained from 3 dogs and processed to generate PBMCs, as noted in Fig. 1. Monocytes were enriched by plastic adherence of PBMC to triplicate wells of a 24-well plates and differentiated to macrophages by incubation in M-CSF, as noted in Methods. Macrophages were ether untreated or treated for 18 h with either 1 ul/ml, 5 ul/ml, 10 ul/ml of LTC or 10 ng/ml canine IFNg or 500 ng/ml LPS. Secretion of TNFα in supernatants was assessed by canine TNFα ELISA (panel **a**). Expression of MHCII was assessed by flow cytometry (panel **b**), as noted in Methods. Data were analyzed using one-way ANOVA with multiple means comparisons. (*, *P* ≤ 0.05, **, *P* ≤ 0.01, *P* ≤ 0.001). These results are representative of a total of 3 separate and independent experiments

To assess the response of macrophages to LTC, monocyte-derived macrophage (MDM) cultures were treated with 0.5, 1.0, 5.0 and 10 μl/ml of LTC for 24 h and supernatants were collected for TNFα analysis by ELISA. In addition, cells were detached and immunostained for flow cytometric assessment of MHCII expression. In Fig. 2, treatment of MDM with increasing dosages of LTC was found to stimulate release of increasing amounts of TNFα (Fig. 2a). In addition, LTC treatment stimulated up-regulation of surface MHCII expression by MDM (Fig. 2b). Higher concentrations of LTC (≥ 10 μl/ml) led to cytoxicity and decreased release of TNFα.

### LTC activate macrophage bactericidal activity

**Fig. 3:**
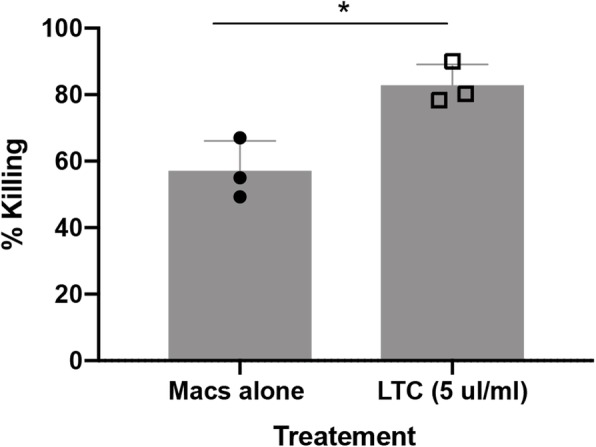
Macrophages activated by LTC exhibit increased bactericidal activity. Monocyte-derived macrophages (MDM) from 3 dogs were treated with LTC for 24 h, after which the cells were infected with a canine methicillin-resistant strain of *S. pseudointermedius* at an MOI of 5 and killing of internalized bacteria was assessed by comparing CFU from macrophage cultures in untreated vs. LTC treated cells. Comparisons between groups were done using ANOVA, followed by Tukey multiple means post-test. Analyses were performed using Prism 8 software (GraphPad, La Jolla, CA). For all analyses, statistical significance was determined if *p* ≤ 0.05

We next evaluated whether macrophage activation by LTC might also be accompanied by induction of bactericidal activity, since activated monocytes and macrophages are likely to interact with bacterial pathogens in the upper respiratory tract. Monocyte-derived macrophages from 3 dogs were treated with LTC for 24 h prior to inoculation with a methicillin-resistant clinical isolate of *Staphylococcus pseudointermedius*, as noted in Methods. Macrophage killing of *S. pseudointermedius* by control and LTC-activated macrophages was evaluated 3 h after infection (Fig. 3). These studies revealed that LTC treatment triggered significant killing of *S. pseudointermedius* by macrophages, indicating that macrophage activation and upregulated MHCII expression was also accompanied by increased bactericidal activity. The macrophage killing activity induced by LTC treatment was comparable to that elicited by IFNγ treatment of macrophages (data not shown).

### LTC adherence and uptake by canine epithelial cells

**Fig. 4:**
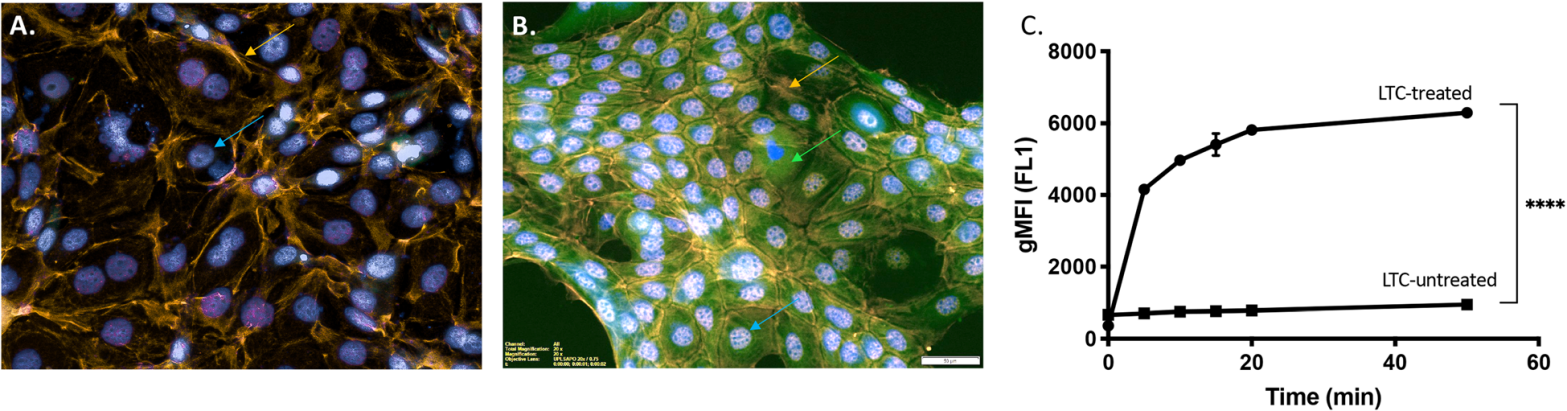
The canine squamous cell carcinoma epithelial cell line CSCAG891 binds rapidly to LTC. A canine squamous cell carcinoma cell line, CSCAG891, was established from an excised canine carcinoma tumor. Cells were grown in 6-well plates to approximately 90% confluence in DMEM 10% FBS. Triplicate cultures of CSCAG891 cells were treated with either nothing (**a**) or 10 μl of TopFluor®-labeled LTCs for 5 min followed by washing 3X in medium (**b**). For microscopy, images of CSCAG891 cells either untreated or treated with 10 μl of TopFluor®-labeled (green arrow) CSCAG cells were subsequently stained with the cross-reactive human epithelial cell marker EpCAM (orange arrows) and nuclei stained with DAPI (blue arrows). For analysis of adherence of LTC to CSAG891 cells, cells were treated with LTC and removed from wells by treatment with trypsin, filtered and assayed by flow cytometric analysis for TopFluor®- stained cells over a period of 50 min, (**c**). Measured green channel (FL1) gMFI for triplicate samples was compared to unstained controls over each time point and differences were analyzed by ANOVA with a Geisser-Greenhouse’s epsilon value of 0.2802. (****, *P* ≤ 0.001)

Studies were done next to assess adherence, uptake and internalization of LTC by dog epithelial cells, since activation of TLR3 and TLR9 only occurs in the intracellular endosomal compartment of cells [16]. To address this question with respect to the assessment of epithelial cells most likely to take up LTC complexes initially in the nose and oropharynx, LTC were fluorescently tagged and incubated with a canine squamous cell carcinoma cell line (CSCAG891) established previously in our laboratory (Fig. 4**).** Nearly confluent cultures of CSCAG891 cells seeded in 6-well plates were incubated with 10 μl LTC labeled with green fluorescent Topfluor®-labeled cholesterol (see Methods) per well for various amounts of time and cellular uptake was assessed by confocal microscopy (Fig. 4a) and flow cytometry (Fig. 4b).

We found that incubation with LTC resulted in significant uptake and intracellular distribution of the LTC complexes (Fig. 4). In addition, flow cytometry revealed rapid uptake of LTC at various time points following in vitro incubation with CSCAG891 cells. The geometric mean fluorescence intensity (gMFI) was measured by flow cytometry and plotted in Fig. 4b. These results establish that LTC are readily taken up and internalized by dog epithelial cells, which predicts that LTC would also undergo uptake by the oropharyngeal and nasal epithelium in vivo.

### Intranasal and oral mucosal administration of LTC results in immune cell infiltration and activation of upper airway and oropharyngeal tissues in dogs

**Fig. 5:**
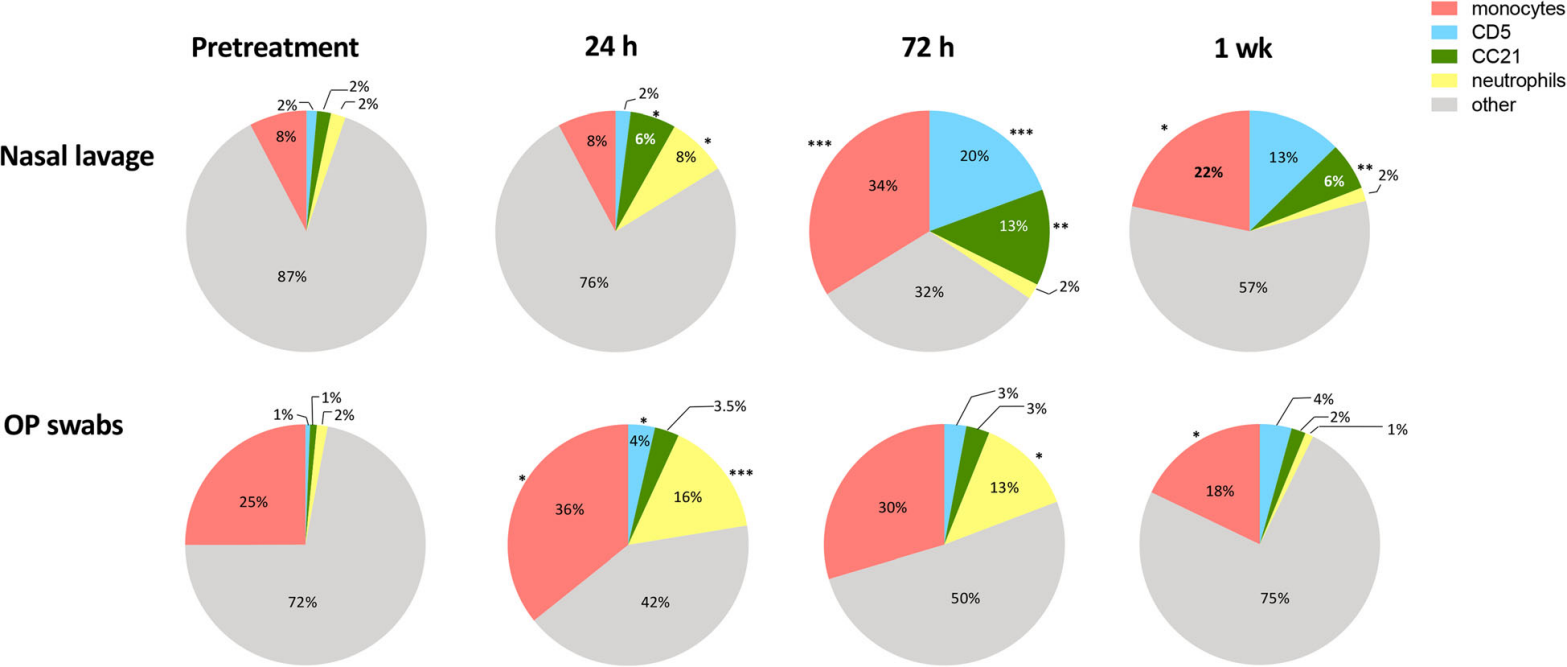
Cellular responses in the nose and oropharynx to mucosal administration of LTC in dogs. Dogs (*n* = 6 per group) were treated intranasally and orally with LTC, as noted in Methods, and nasal lavage and oropharyngeal swab samples were obtained pre-treatment and again at 24 h, 72 h, and 7 days to assess changes in immune cell populations (T cells, B cells, monocytes, and neutrophils) over time, using flow cytometry as noted in Methods. Pie charts were generated to illustrate changes in immune cell composition in nasal and oropharyngeal (OP) regions over time. Asterisks indicate significant changes in cellularity when compared to pretreatment values. Comparisons between 3 or more groups were done using ANOVA, followed by the Tukey multiple means post-test. Analyses were performed using Prism 8 software (GraphPad, La Jolla, CA). (*, *P* ≤ 0.05; **, *P* ≤ 0.01; ***, *P* ≤ 0.005; ****, *P* ≤ 0.001)

Healthy adult dogs (*n* = 6 per group) were treated with a single administration of LTC, delivered by both the intranasal and oral routes, as described in Methods. Cellular responses to LTC administration were evaluated over time in treated animals. Nasal lavage and oropharyngeal swab samples were obtained prior to treatment, and at 24 h, 72 h and 7 days after treatment. Cells from the nasal cavity and oropharynx were obtained by nasal lavage and swabbing the oropharynx, respectively (Fig. 5). Cells obtained from the two sites were analyzed by flow cytometry. T lymphocytes are identified as CD5^+^ cells, B cells were identified as CD21^+^ cells, monocytes were identified as CD11b^+^ and CD14^+^ positive cells, and neutrophils were identified based on typical forward versus side-scatter characteristics.

The percentages of each cell type in nasal lavage samples over time were plotted as pie chart diagrams, as noted in Fig. 5**.** For nasal lavage samples, compared to pre-treatment samples, samples obtained following LTC treatment had significant increases in the percentage of CD5^+^ T cells at 72 h and 7 days post treatment. The percentage of monocytes was increased significantly in nasal lavage fluid, from 8% of pre-treatment lavage cells to 34% of lavage cells at 72 h and remained elevated at 22% after 7 days. The percentage of B cells significantly increased (from 2 to 6%) in nasal lavage samples at 24 h after LTC treatment and further increasing to 13% at 72 h and remained elevated at 6% at 7 days post treatment. Neutrophils in nasal lavage fluid initially rose from 2 to 8% at 24 h after LTC treatment but declined to pre-treatment levels in 72 h remaining at pre-treatment percentages at 7 days.

Cell samples obtained by swabs from the oropharynx (OP) had modest but significant increases in T cells following LTC treatment, increasing from 1% at pre-treatment levels to 4% 24 h post-treatment and remaining between 3 and 4% of oropharyngeal cells at 72 h and 7 days post-treatment (Fig. 5). The percentages of monocytes increased from 25 to 36% at 24 h and then returned to near or below pre-treatment values at 72 h and 7 days following LTC administration. The average percentage of B cells modestly increased in the oropharynx 24 h after LTC treatment from 1 to 3.5%, remained near those levels at 72 h post treatment and decreased to 2% by 7 days. Neutrophils increased from 2 to 16% after 24 h and declined to 13% in 72 h and returned to pre-treatment levels 7 days after LTC administration.

### Monocytes from both nasal lavage and oropharyngeal samples exhibit increased expression of MHCII 1 week after LTC treatment

**Fig. 6:**
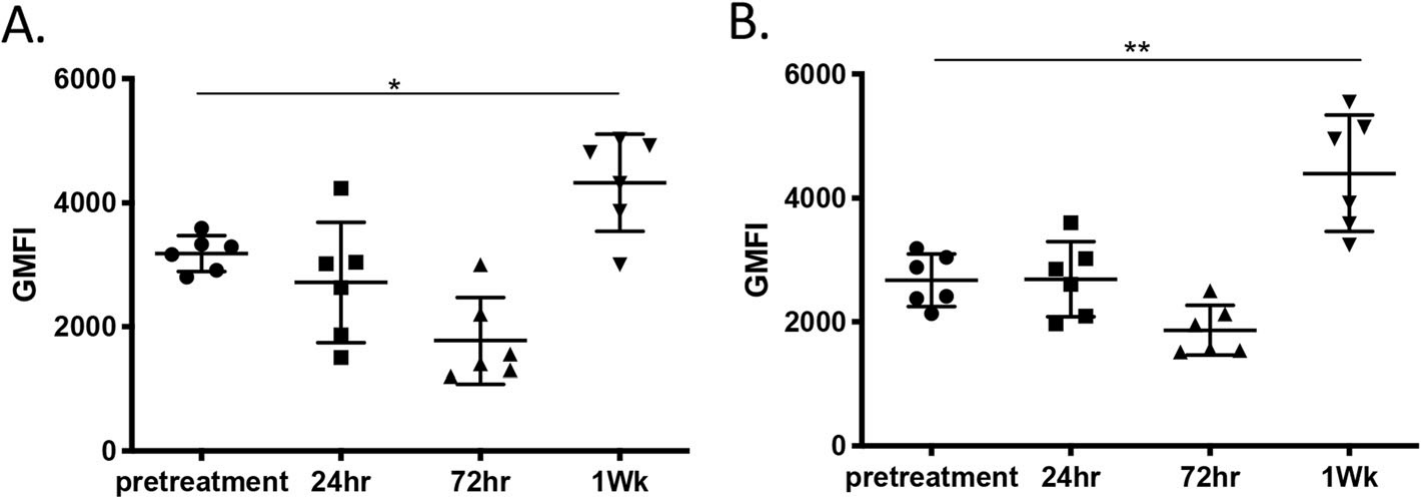
MHCII expression by monocytes in nose and oropharynx to LTC treatment. Nasal lavage and oropharyngeal swab samples were collected from dogs (*n* = 6) following treatment with LTC, and immunostained for analysis by flow cytometry, as noted in Methods. Monocytes from nasal lavage (**a**) and oropharyngeal (**b**) specimens were analyzed for expression of MHCII, prior to treatment and again at 24 h, 72 h, and 7d following LTC administration. Data were presented as geometric mean fluorescent intensity (gMFI) and analyzed using one-way ANOVA with multiple means comparisons. (*, *P* ≤ 0.05, **, *P* ≤ 0.01)

Up-regulation of MHCII expression is a key phenotypic feature of activated monocytes [26] . Therefore, MHCII expression by monocytes obtained pre-treatment, and 24 h, 72 h and 7 days after LTC administration was determined by flow cytometric analysis. This analysis revealed that MHCII expression was significantly upregulated on monocytes isolated from both nasal lavage fluid (Fig. 6a) and oropharyngeal swabs (Fig. 6b) 7 days after LTC treatment, indicative of immune activation following LTC administration. The upregulation of MHCII expression by monocytes following in vitro LTC treatment was also observed (see Fig. 2). These data indicate therefore that LTC treatment upregulated MHCII expression by monocytes, most likely by inducing production of IFNγ and TNFα.

**Fig. 7:**
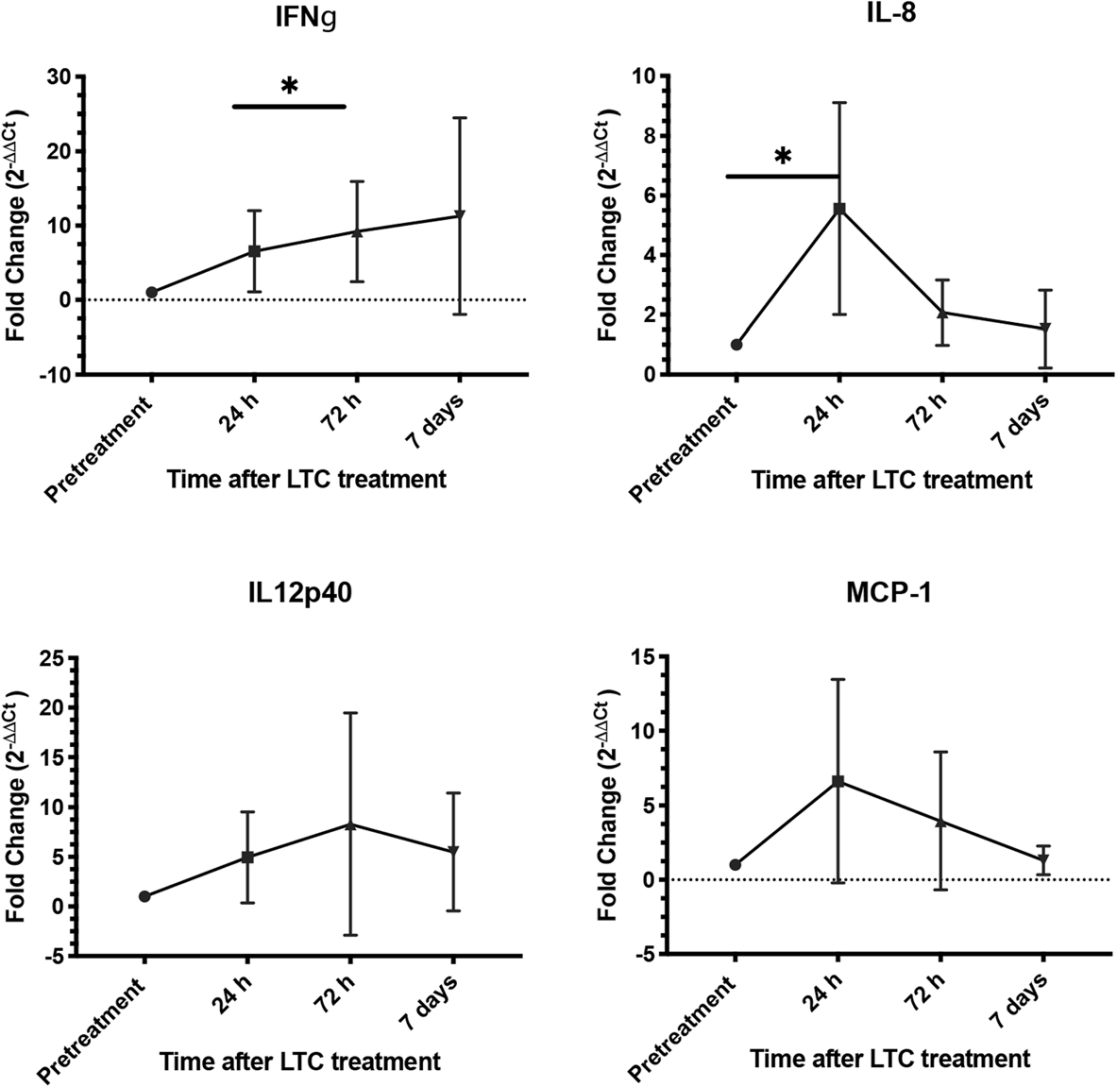
Induction of inflammatory cytokine gene expression in oropharynx of dogs following mucosal administration of LTC. Samples were obtained from oropharyngeal swabs of dogs (*n* = 6) before and after treatment with LTC. At the indicated time points, RNA was extracted from swab samples and expression of 4 key inflammatory cytokine genes (IFNγ, IL-8, IL-12, and MCP-1) was assessed using qRT-PCR, as noted in Methods. Data were expressed as fold-change in mRNA transcript levels over time. Significance was determined using the Wilcoxon signed-rank test, with *, *P* ≤ 0.05

To assess the impact of LTC treatment on expression of other inflammatory cytokines, RNA was extracted from cells obtained by nasal swab or oropharyngeal swabs obtained pre-treatment and on day 1, day 3, and day7, and subjected to *q*RT-PCR to assess expression of IFNγ, IL-8, IL-12p40, and MCP-1 (Fig. 7). These studies revealed that LTC treatment triggered significant upregulation of expression of mRNA for two key innate immune cytokines (IFNγ and IL-8) along with numerical increases in expression of mRNA encoding IL-12 and MCP - 1. These results are consistent with broad, local induction of key anti-viral and anti-bacterial cytokines in upper airway tissues following topical administration of LTC in healthy dogs.

### Non-specific induction of anti-herpesvirus immunity by LTC treatment

**Fig. 8:**
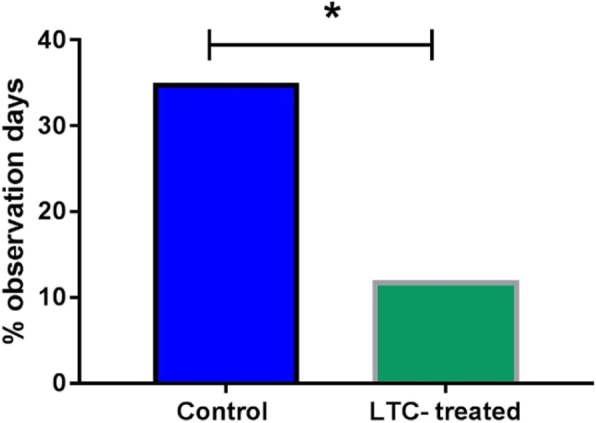
Non-specific induction of antiviral immunity in dogs by LTC treatment. Purpose bred beagles (*n* = 21) shortly after exposure to canine herpesvirus were randomly assigned to treatment groups, with *n* = 7 receiving a single intranasal and oral LTC treatment, and *n* = 14 being treated with PBS, as noted in Methods. Dogs were monitored for clinical signs of herpesvirus infection, including conjunctivitis scores, as noted in Methods. The overall mean conjunctivitis score for each group was calculated and plotted. Statistical significance was evaluated by Fisher’s exact test (* = *p* < 0.05). The majority of clinical signs were recorded in the first 7 days after initiation of treatment (94%) and none of the dogs had clinical signs consistent with canine herpesvirus after Day 13

The preceding studies indicated that mucosal delivery of LTC to healthy dogs triggered local innate immune activation. Prior studies in rodent and cat models also reported that LTC treatment administered intranasally elicited strong antiviral activity [22–24]. Therefore, we used the opportunity provided by an inadvertent canine herpesvirus outbreak in 21 research Beagle puppies to evaluate the potential effectiveness of LTC as an early immunotherapy (see the Materials and Methods). Nucleic acids from canine herpesvirus, but not from the other canine viruses that were screened, were amplified from 20 of the 21 puppies. Based on this finding, a diagnosis of canine herpesvirus infection was made. Conjunctivitis was the most consistent clinical finding in affected dogs. The puppies were housed in 3 rooms, each containing clinically ill and PCR positive puppies. The LTC treatment was administered once to each of 7 puppies (0.5 ml LTC per nostril and 2 ml orally), while 14 puppies were maintained as untreated controls. The proportions of observation days (40 days in total) in which treated animals (13.3% of total observation days) and control, untreated dogs (35% of total observation days) had evidence of conjunctivitis were calculated and compared (Fig. 8). The difference in conjunctivitis positive observation days was statistically significant when LTC-treated and control dogs were compared and suggested that a single administration of LTC induced a therapeutic response consistent with induction of early anti-viral immunity.

### Impact of LTC treatment on the oropharyngeal microbiome

The microbiome of the upper respiratory tract and GI tract is known to be relatively stable over time in healthy individuals but can be perturbed significantly by administration of antimicrobial drugs [27–29]. However, much less is known about the impact of local activation of innate immune responses on the composition of the microbiome [30, 31]. For example, it is possible that local activation of immune defenses, including cytokines derived from epithelial cells and immune cells, could significantly alter the composition of the bacterial flora by either depleting or expanding certain bacterial populations. Alternatively, it is possible that the local microbiome may be relatively resistant to such immune perturbations. To address this question, the overall composition of the microbiome of the oropharynx in dogs (*n* = 6) that were treated with LTC was determined by 16S RNA sequencing immediately prior to treatment and again at 7 days and 14 days after treatment. Oropharyngeal samples were obtained by swabs and extracted microbial DNA was sequenced using an Illumina platform (Novogen, San Diego, CA). Data were analyzed as described in Methods.

**Fig. 9:**
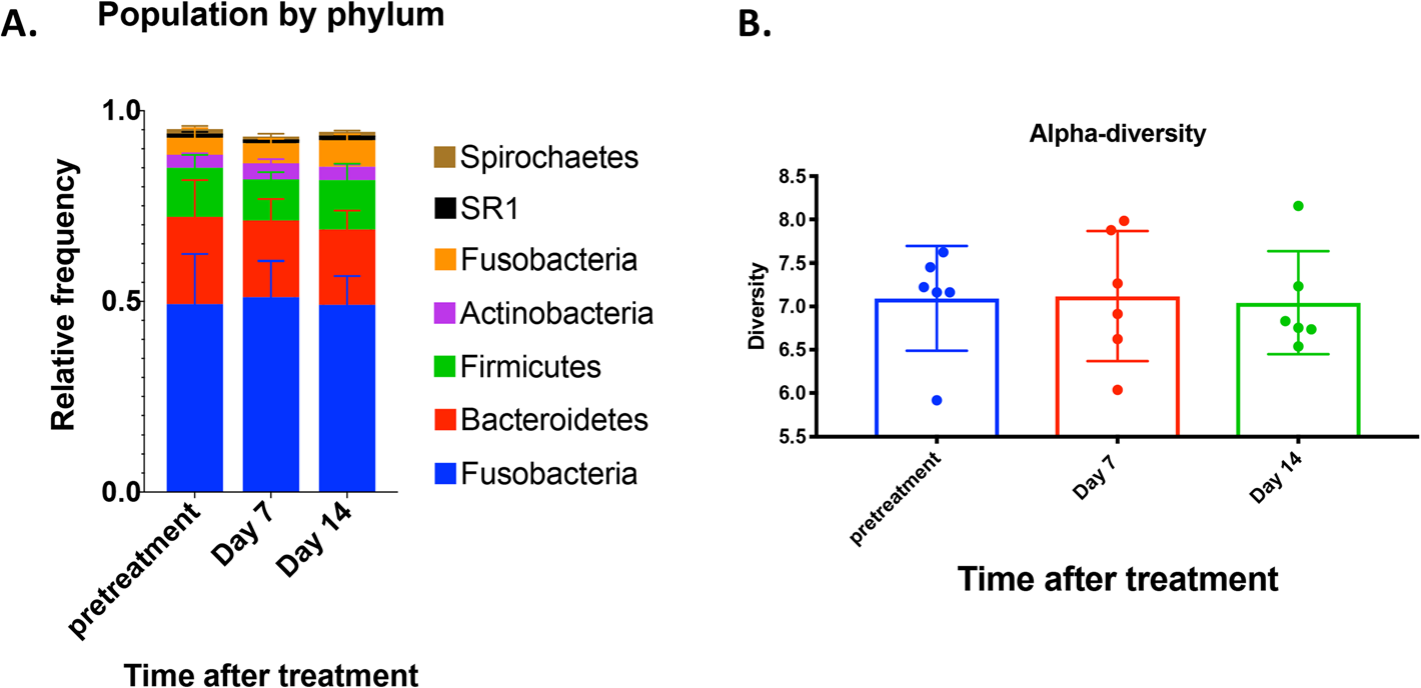
Impact of LTC administration on oropharyngeal microbiome of dogs. Healthy dogs (*n* = 6) were treated by intranasal and oral administration of LTC, and oropharyngeal swabs were collected for evaluation of the resident microbiome, using 16S sequencing, as described in Methods. At the treatment times indicated, the relative abundances of key major phyla (*Spirochaetes, Fusobacteria, Actinobacteria, Firmicutes, Bacteroidetes, and Fusobacteria)* were determined by 16S sequencing and represented in panel (**a)**. In panel (**b)**, the alpha-diversity of all 6 phyla were compared over a 2-week period (**b**). These analyses did not detect significant differences in the phyla composition, or in the alpha diversity, as assessed by ANOVA

Based on the results of 16S RNA sequencing (Fig. 9), it was apparent that the major phyla of bacteria located in the oropharynx of dogs did not change in any meaningful way following mucosal delivery of LTC. For example, significant changes in the relative abundance of major bacterial phyla including *Spirochaetes, Fusobacteria, Actinobacteria, Firmicutes, Bacteroidetes, and Fusobacteria* (except on day 14 for Fusobacteria) were not observed when pre-treatment abundances were compared to day 7 or day 14 post treatment abundances. Thus, while LTC administration transiently activated innate immune responses within the oropharyngeal region of dogs, the microbiome of the oropharynx appeared to be relatively resistant to perturbation by immune stimuli. These findings suggest that the local microbiome in healthy animals is relatively resistant to perturbation by the local effects of innate immune activation.

## Discussion

Despite advances in vaccine technologies and availability, there remains an unmet need for new non-specific immunotherapeutics for dogs for prophylaxis or early therapy of infectious diseases. To address this need, we describe here a new mucosally active immunotherapy that has the potential to be used to prevent or treat early viral or bacterial infections or to treat established infections where strong induction of type I innate immune responses may lead to pathogen clearance. Moreover, there is also evidence that strong activation of innate immune responses and IFNγ production can synergize with conventional antibiotics to clear chronic, intracellular bacterial pathogens. For example, we demonstrated previously that activation of innate immunity with LTC in rodent models of *Burkholderia* and *Francisella* infection significantly augmented the effectiveness of antibiotic therapy [20, 21].

Our in vitro studies with LTC revealed strong activation of production of key innate immune cytokines, including IL-8, MCP-1, IL-12p40, IFNγ and TNFα following LTC treatment in dog PBMC cultures and oropharyngeal cells (see Figs. 1, 2 and 7). We also found that LTC were taken up and internalized efficiently by canine epithelial cells following in vitro incubation (Fig. 4). The ability to adhere to and enter epithelial cells is important, because sustained contact with epithelial cells and immune cells in the nasal cavity and oropharynx is important for LTC to be internalized, engage TLR3 and TLR9 receptors in the endosomal compartment, and to activate sustained innate immune responses. Importantly, we also demonstrated that application of LTC to mucosal surfaces of the upper airways and oropharynx of dogs elicited local immune activation, as reflected by an increase in leukocyte recruitment and activation of cytokine production (see Figs. 5 and 6).

Infiltration of monocytes and lymphocytes into both the nasal cavity and the oropharynx provided direct evidence of LTC stimulation of local innate immune activation and chemokine production. Activation of cytokine production was also confirmed by RT-PCR analysis (see Fig. 7). In both the nose and the oropharynx of dogs, the cellular infiltration generally peaked at 72 h after LTC administration. Overall cellularity remained elevated in the nose for at least 7 days (the last time point evaluated), whereas cell counts returned to normal in the oropharynx by 7 days. Leukocyte infiltration was much more robust in the nose, and consisted of strong increases in T cells, monocytes, and B cells, with relatively smaller increases in neutrophils. In contrast, cellular infiltration in the oropharynx was dominated by myeloid cells (monocytes, neutrophils) with smaller T and B cell responses. These differences most likely reflect local differences in chemokine production in response to TLR stimulation, as well as heterogeneity in resident tissue cell populations. Overall, the enhanced leukocyte infiltration into upper airway tissues following LTC administration could enhance both antiviral and antibacterial immune defenses, via direct mechanisms of control (e.g., macrophage bactericidal activity, see Fig. 3) or via indirect mechanisms such as cytokine and antibody production.

We also demonstrated that LTC treatment induced therapeutic anti-viral immunity in dogs when administered early after canine herpesvirus infection. For example, in dogs infected in a canine herpesvirus outbreak, we found that early mucosal administration of LTC generated significant reduction in clinical signs (e.g., conjunctivitis) associated with canine herpesvirus infection (see Fig. 8). Similar results were also observed recently in cats treated with LTC shortly prior to intranasal challenge with FHV-1, where a significant reduction in clinical signs of infection and viral shedding was observed [22, 24]. We have also observed complete or nearly complete anti-viral and anti-bacterial protection in a number of different lethal rodent models of infection following intranasal administration of an earlier version of LTC [20, 21, 23]. Thus, it is apparent that LTC mucosal immunotherapy can elicit significant antiviral immunity in at least 3 different animal species (mouse, dog, cat).

## Conclusion

In summary, we have demonstrated using in vitro and in vivo studies that a new immunotherapeutic consisting of liposomes complexed to TLR3 and TLR9 agonists potently activated innate immune responses in dogs. The immune responses triggered by intranasal and oral administration were primarily concentrated locally, based on evidence of cellular immune infiltrates at the sites of administration and the lack of systemic responses (e.g., fever) to treatment. These LTC immune complexes have potential utility as a new method of rapidly eliciting non-specific protective immunity in the event of an outbreak (e.g., canine influenza virus or *Bordetella* infections in boarding facilities) while potentially also having a direct translational utility against infections in humans and other species. Moreover, concurrent administration of LTC with conventional vaccines may improve or accelerate overall vaccine immunity. Finally, increasingly veterinarians will be encouraged to seek non-antimicrobial alternatives to disease prevention or therapy, and the use of potent, mucosally active immunotherapeutics such as LTC are likely to have an important role to play in these new approaches.

## Methods

### Preparation of liposome-TLR complexes (LTC)

Liposomes were prepared by drying cationic lipid 1,2-dioleoyl-3-trimentylammonium-propane (DOTAP) and cholesterol (Avanti Polar Lipids, Alabaster, AL) to a thin film on glass tubes, using a vacuum desiccator. The lipids were then rehydrated to form liposomes, as described previously [16, 24, 32]. Polyinosinic, polycytidylic acid (pIC; InVivoGen, San Diego, CA) and non-coding commercial plasmid DNA (PCR2.1, Life Sciences, CA) were added to the liposomes to form liposome-TLR agonist complexes (LTC), as reported previously [16, 24, 32]. The final concentration of both TLR agonists were 25 μg/ml. The endotoxin content of the plasmid was < 0.25 EU/ug and pIC was certified to be LPS-free. For in vitro tracking studies, liposomes were formulated to contain 10% (v/v) TopFluor-labeled cholesterol (Avanti, Alabaster, AL).

### In vitro cell activation with LTC

To assess cell activation, peripheral blood mononuclear cells (PBMC) from healthy dogs were prepared as described previously [33]. Briefly, whole blood was obtained by jugular venipuncture and collected into EDTA tubes, then diluted 1:2 with sterile PBS, layered over a Ficoll (GE Healthcare, Uppsala, Sweden) gradient and centrifuged for 30 min. Following centrifugation, PBMC were collected from the Ficoll interface and washed twice in PBS and then re-suspended in complete tissue culture medium consisting of DMEM (Thermo Fisher Scientific, Watham, MA) medium containing 10% FBS (VWR Seradigm, Denver, CO), essential and non-essential amino acids, penicillin and streptomycin (Gibco-Thermo Fisher Scientific, Pittsburgh, PA). After counting, cells were plated in 96-well flat bottom plates (Celltreat, Pepperell, MA) at a density of 1 X 10^6^ cells/well in 200 μl medium. For PBMC activation, LTC were added at 4 different dilutions (0.02 μl/well, 0.2 μl per well, 1 μl per well, and 2.0 μl per well) in triplicate wells of PBMC in 200 μl complete DMEM, with careful mixing, and the cells were then incubated for an additional 24-48 h. Conditioned medium was collected for IFNγ and TNFα assays and cells analyzed via flow cytometric analysis for modulation of activation markers (see below). PBMC were assayed at least twice using separate donor animals.

### Generation of monocyte-derived macrophages

To generate monocyte-derived macrophages (MDM) in vitro, PBMC were plated in 24-well plates at a density of 5 X 10^6^ cells/ml and allowed to adhere for 4 h, after which non-adherent cells were removed by gentle washing with PBS. The adherent cells were then cultured in complete DMEM with the addition of 10 ng/ml recombinant human M-CSF (R&D systems,) for 7 days, and replacing with fresh M-CSF medium every 3 days. This technique produced nearly pure cultures of dog macrophages, as assessed by flow cytometry and immunostaining for CD11b expression (data not shown).

### Analysis of cytokine gene expression by qRT-PCR

Samples were obtained from oropharyngeal swabs of dogs (*n* = 6) before and after treatment with LTC, and expression of IL-8, MCP-1, IL-12p40, and IFNγ genes was determined via quantitative real time-(qRT)-PCR, using previously published primers [34, 35]. Briefly, cDNA was prepared by isolation of RNA followed by reverse transcription using a commercial kit (Qiagen, Germantown, MD) followed by amplification using SYBR™ green primers (Bio-Rad, Hercules, CA). Amplification was performed using a qPCR MX3000p system instrument (Agilent, Santa Clara, CA). All primers were validated to have an efficiency > 90% using stimulated and unstimulated healthy dog PBMC. pRT-PCR was used to quantify cytokine transcript levels as shown previously [24].

### IFNγ and TNFα ELISA

Supernatants from PBMC cultures were analyzed for IFNγ and TNFα using commercial canine IFNγ (DuoSet® Canine IFNγ kit; R&D systems, Minneapolis, MN) and TNFα (DuoSet® Canine TNFα kit; R&D systems, Minneapolis, MN) according to manufacturer’s protocols.

### Flow cytometry for analysis of in vitro activated cells and nasal and oropharyngeal lavage specimens

Cells cultured in vitro were harvested after 24 h of LTC stimulation and immunostained with fluochrome-conjugated antibodies: T cells: CD5-PE; CD4-PB, CD8-APC; B cells: MHCII-FITC, CD21-APC and CD45-PB; monocytes: MHCII-FITC, CD14-APC, CD11b-PC7, CD45-PB. Prior to adding staining antibody, cells were preincubated for 5 min with normal dog serum containing human IgG and anti-mouse FcRIII antibodies to block any non-specific antibody binding. Cells were subsequently immunostained with the conjugated antibodies for 20 min at 4 °C in FACs buffer (PBS with 2% FBS and 0.05% sodium azide).

Nasal and oropharyngeal samples obtained by gentle swabbing of the dogs (see below) were transferred to 50 ml conical tubes containing 25 ml of sterile PBS and the sampling swabs were used to gently stir the solution to dislodge cellular material from the swabs. The solution was then filtered through 50 μm cell strainers (Corning, Fairport, NY) and pelleted by centrifugation. Cells from the resuspended pellet were washed once with PBS then stained directly with conjugated antibodies described above. Additional aliquots of cells were stained with isotype-matched antibodies as controls for each primary antibody. Flow cytometric analysis was performed using a Beckman Coulter Gallios flow cytometer (Beckman Coulter, Indianapolis, IN), and data were analyzed using FlowJo software (Tree Star, Ashland, OR).

### Animal studies

All animal studies were approved by the *Institutional Animal Care and Use Committee* (IACUC) at a contract research facility in Fort Collins, CO (Protocol # 170024). Purpose-bred Beagle dogs used for these the studies were purchased from a commercial vendor. Dogs were housed in large kennels at the facility under standard light vs. dark conditions and cared for by qualified animal care technicians. Following completion of the studies, all dogs were adopted to local owners in the Ft. Collins area.

### Administration of liposome-TLR complexes to study animals

A power calculation indicated that the minimum number of dogs to be used in this study was *n* = 6 for each group. To minimize the number of research animals subjected to treatment, we chose to examine a total of 12 dogs: Group 1: 6 untreated and Group 2: 6 LTC-treated. Study dogs (*n* = 6 per group) were treated by a single administration of LTC by the intranasal and oral routes. For LTC-treated dogs, 0.5 ml LTC was administered to each nostril, using a 1 ml syringe and minimal manual restraint. In addition, each dog also received 2.0 ml of LTC administered orally, directed to the back of the throat, using a 3 ml syringe. Control animals (*n* = 6) were administered sterile PBS, 0.5 ml per nostril and 2.0 ml orally. The intranasal dose of each TLR ligand for each dog was approximately 20 μg per kg body weight.

### Collection of nasal lavage and oropharyngeal samples

To collect nasal lavage samples, dogs were briefly restrained manually in a head-down position, and 5 ml of pre-warmed sterile PBS solution was quickly administered into each nostril, and the fluid backflow was collected from the nostrils in 15 ml conical tubes. PBS was administered once more and pooled fluid from each dog was stored on ice. Oropharyngeal samples were collected by gently swabbing the caudal oropharyngeal region by gently rubbing and rolling against the mucosa. Swabs were placed in 15 ml conical tubes containing complete DMEM tissue culture medium containing 10% FBS and stored on ice. Both Nasal and oropharyngeal samples were collected 24 h prior to administration of LTC and samples were likewise obtained again at 72 h and 7 days post-LTC treatment.

### Preparation and analysis of nasal and oropharyngeal samples

Nasal lavage fluid was diluted with 3 ml of complete DMEM and oropharyngeal swabs were collected from the distal oropharynx and immediately placed in 2 ml of complete tissue culture medium in 15 ml polypropylene tubes on ice. To dislodge cells from the swabs, the samples were lightly vortexed. Fluid was subsequently transferred to a new tube and the swabs were again rinsed with 2 ml of PBS and pooled with the first wash. Pooled samples were filtered through a 70 μm cell strainer (CellTreat, Pepperell, MA) to remove large debris and mucus. Samples were then centrifuged and the pellets were resuspended in 0.5 ml of PBS and 10 μl aliquots were stained with 0.4% trypan blue and blue-excluding cells were counted using a Nexcelom Cellometer™ Auto T4 cell counter (Nexcelom; Lawrence, MA). Nasal lavage samples were processed similarly. To determine cell types and activation states, equivalent numbers of cells were processed and stained for flow cytometric analyses as described above.

### Non-specific induction of anti-herpesvirus immunity by LTC treatment

Beagle puppies (*n* = 21) that were originally purchased for a separate study but developed sneezing and conjunctivitis had orophargygeal swabs collected as described. The samples were assessed by a PCR panel performed at a commercial laboratory (Antech Diagnostics, Lake Success, NY) and the only viral nucleic acids of common canine pathogens that were amplified were those of canine herpesvirus (detected in 20 of 21 animals). The affected 21 dogs were randomly allocated to housing in 3 different rooms of 7 dogs each. Each room contained clinically ill and canine herpesvirus PCR positive dogs. Within 24 h of the first recognized clinical signs, 7 dogs were administered 0.5 ml LTC per nostril and 2 ml orally, while 14 dogs were maintained as untreated controls. Two trained and study-blinded observers then applied a standardized clinical score rubric to dogs in each room for 30 min each day for 40 days. The primary clinical sign noted over the course of the study was conjunctivitis. The proportions of 40 total observation days in which treated and control dogs had evidence of conjunctivitis were calculated and compared by means of Fisher’s exact test (Fig. 8).

### 16S sequencing of bacterial microbiomes in oropharyngeal samples

Oropharyngeal swabs from dogs were collected prior to LTC treatment, in addition 24 h, 72 h and 7 days post treatment. Bacterial pellets collected from swabs were concentrated by high speed centrifugation stored in PBS at − 20 °C until processing for DNA extraction. Microbial DNA extraction was performed using a MoBio Powersoil DNA Isolation kit (Qiagen, Valencia, CA) according to manufacturer’s protocol. 16S rRNA sequencing was performed by Novogene (Chula Vista, CA). Negative controls were verified on Nanodrop 1000 to have < 2 ng/uL of total DNA. DNA concentration and purity was monitored on 1% agarose gels.

The ribosomal RNA genes of the bacterial V4 region were amplified using V4: 515F-806R in accordance with the Earth Microbiome project [36]. All amplification reactions were carried out in Phusion High-Fidelity PCR Master Mix (New England Biolabs, MA). PCR products, sequencing libraries library quality and sequencing were generated, assessed and performed respectively as described in [37]. Sequence quality control, adapter trimming and feature table construction were performed according to the QIIME2 version 2018.2 demux summarize DADA2 [38].

Operational Taxonomic Units (OTUs) were resolved at 97% sequence similarity using QIIME as described in [37, 39]. For taxonomic assignment, the Greengenes 16S database was used at 0.8 confidence level. Phylogenetic tree was constructed using Qiime2 phylogeny fasttree [40]. Alpha diversity and beta diversity (weighted and unweighted unifractions) was calculated using Qiime2 diversity core metrics [41]. Differential abundance testing was performed using Analysis of Composition of Microbiomes (ANCOM) [42]. Significance in relative abundance on Phylum, Class, Order, Family and Genus levels was calculated using 2-way ANOVA with Tukey post-test. Graphical results were plotted using Graph Pad Prism 8 (GraphPad Software, La Jolla California USA).

### Confocal microscopy and cell imaging

To assess uptake of LTC by relevant target cells in the nasal cavity and oropharynx (ie, squamous epithelium and macrophages), LTC were incubated with canine squamous cell carcinoma cells (cell line CSCAG891, generated in the Dow lab) and with canine macrophages (not shown). Cells were incubated with serial dilutions of LTC prepared with a fluorescent liposome (TopFluor, Avanti Polar Lipids, Alabaster, AL) for tracking. After the indicated periods of incubation, the cells were fixed and then imaged using an Olympus (Waltham, MA) IX3 confocal microscope. Images were processed and analyzed using Olympus CellSens® software.

### Macrophage killing assays

Monocyte-derived macrophages (MDM) were derived as described above and were plated at a density of 1 X 10^5^ cells per well in 48-well plates and subsequently either left untreated or treated with LTC at 5 μl/ml or 10 ng/ml canine IFNγ (data not shown in Fig. 3) in 500 μl for 24 h. Untreated or treated MDM were infected at MOI =5 in HBSS containing Ca^++^ and Mg^++^ in 10% dog serum for 1 hr. To enumerate intracellular concentrations of bacteria, MDM were lysed with sterile distilled water and diluted 10-fold serially in PBS and plated on quadrants of brain heart infusion (BHI) plates. Some cultures were stopped immediately after infection to determine initial intracellular bacterial concentrations, while others were incubated for an additional 2 h to allow for bacterial killing prior to enumeration of remaining intracellular bacterial concentrations. Bacterial colony forming units (CFU) were determined and CFU from the 1 h incubation was compared to the 2 h incubation to determine intracellular killing efficiency. The remaining CFU after 2 h was divided by the CFU from the 1 h incubation and this quotient was multiplied by 100 represented the percent killing of MRSP by the treated or untreated MDM.

### Statistical methods

For comparisons between data sets with two treatment groups, statistical significance was evaluated by Fisher’s exact test (* = *p* ≤ 0.05). ANOVA followed by Tukey multiple means post-test was used to perform analyses comparing 3 or more groups.

Statistical significance defined as *p* ≤ 0.05 (*). Analyses were done using Prism 8 software (GraphPad, La Jolla, CA).

## Data Availability

All data generated and/or analyzed during this study are included in this published article. Microbiome data has been uploaded to the European Nucleotide Archive (ENA) (primary accession # PRJEB34170) https://www.ebi.ac.uk/ena/submit/sra/#studies.

## Abbreviations

LTC: Liposome TLR complexes
MDM: Monocyte-derived macrophages
MHCII: Major histocompatibility complex class II
MRSP: Methicillin-resistant *Staphlococcus pseudintermedius*
